# Impact of oncoplasty in increasing breast conservation rates Post neo-adjuvant chemotherapy

**DOI:** 10.3389/fonc.2023.1176609

**Published:** 2023-09-07

**Authors:** Chaitanyanand B. Koppiker, Devaki A. Kelkar, Madhura Kulkarni, Shweta Kadu, Mugdha Pai, Upendra Dhar, Chetan Deshmukh, Beenu Varghese, Vaishali Zamre, Nutan Jumle, Nutan Gangurde, Anjali Joshi, Rohini Unde, Rituja Banale, Namrata Namewar, Pooja Vaid, Laleh Busheri, George Thomas, Smeeta Nare, Jerome Pereira, Sunil Badve

**Affiliations:** ^1^ Prashanti Cancer Care Mission, Pune, India; ^2^ Center for Translational Cancer Research, a Joint venture between Prashanti Cancer Care Mission and Indian Institute of Science Education and Research (IISER), Pune, India; ^3^ Department of Onco-Sciences, Jehangir Hospital, Pune, India; ^4^ International School of Oncoplasty, Pune, India; ^5^ Orchids Breast Health Centre, A Prashanti Cancer Care Mission (PCCM) Initiative, Pune, India; ^6^ Ashoka University – Department of Biology, Ashoka University, Haryana, India; ^7^ Norwich Medical School, University of East Anglia, Norwich, United Kingdom; ^8^ Department of Pharmacology and Chemical Biology, Emory University, Atlanta, GA, United States

**Keywords:** neoadjuvant chemotherapy, breast cancer, breast conservation for large tumors, mastectomy, oncoplastic breast conservation, frozen section analysis, tumor localization

## Abstract

**Introduction:**

The essential goal of neoadjuvant chemotherapy (NACT) is to downstage the primary tumor making it amenable for breast conservation surgery (BCS). However, since the safety of this surgery is paramount, post-NACT breast conservation rates remain low. As per the recommendation of the 2018 Early Breast Cancer Trialists’ Collaborative Group (EBCTCG) overview of long-term post-NACT follow-up, we have devised a protocol for imaging, localization, rad-path analysis, and documentation of radiotherapy techniques to ensure the safety of post-NACT breast conservation.

**Methods:**

This is a retrospective cohort of 180 breast cancer patients who received NACT and were operated on by a single surgical oncologist from 2015 to 2020. After selection based on published guidelines, patients were treated with neoadjuvant systemic (chemo or hormone) therapy. In cases where primary tumors responded and reduced to 1–2 cm in size mid-NACT, the residual tumors were localized by clips under ultrasound guidance and calcification was wire localized. All patients were treated using appropriate surgical and oncoplastic techniques where indicated. Negative margins were ensured by intra-operative rad-path analysis. Adjuvant chemotherapy and radiotherapy were given as per protocol.

**Results:**

In 81 cases that required mastectomy at presentation, we were able to achieve a 72.8% post-NACT BCS rate with the help of oncoplasty. Overall, 142 of 180 (80%) patients were treated with breast conserving surgery of which 80% (121 of 142) were oncoplasty. Margins were assessed on intra-operative frozen and re-excised in the same setting. No positive margins were reported in final histopath of 142 breast conservation procedures. Post-operative complication rates after breast conservation in the first year were at 17% (24 of 142 including two major complications). Patient reported outcomes were satisfactory with increased satisfaction for breast conservation compared with immediate breast reconstruction.

**Discussion:**

Employing oncoplastic breast surgery (OBS) techniques following stringent protocols for accurate localization of the residual tumor, intra-operative rad-path analysis, and adjuvant treatments, we show successful breast conservation in 72.8% of our mastectomy-qualified patients after downstaging by NACT. We also report satisfactory outcomes for post-NACT surgery, patient-reported satisfaction, and survival.

## Introduction

Breast cancer is the most diagnosed cancer and the leading cause of cancer-related death in India and globally(1, 2). Population-based screening in Western Europe and the USA has enabled early diagnosis, making early intervention possible. With the acceptance of conventional breast conservation surgery (BCS) as a safe technique (3), it has become possible to reduce or completely avoid mastectomy in breast cancer patients, leading to better quality of life (4–6). BCS is now becoming the gold standard of surgical treatment for early breast cancer (7–9). Locally advanced breast cancers (LABCs) and large operable breast cancers (LOBC) are treated with neoadjuvant therapy (NACT) with the aim to downstage these advanced cancers loco-regionally. Downstaging helps to avoid mastectomy in favor of BCS where oncologically and esthetically feasible (9–11).

Conventional BCS is limited to selected patients where it has been shown to be oncologically safe and demonstrable esthetically superior outcomes (12). A hurdle in expanding BCS to patients outside, this limited set is the extent to which post-NACT excision volumes can safely be minimized (13). The Early Breast Cancer Trialists’ Collaborative Group (EBCTCG) overview of long-term adjuvant and neoadjuvant therapy outcomes (14) showed increased loco-regional recurrence rates for post-NACT BCS as compared with mastectomy in long-term follow-up. This overview created more doubts about the safety of post-NACT BCS even though the authors accepted that there were several flaws in the studies included in the overview (discussed later). A combination of these factors has resulted in the slow uptake of post-NACT BCS despite adequate data that show post-NACT BCS to be safe (15). The conversion rates for mastectomy to BCS post-NACT downstaging have therefore remained low (~40%) (14, 16, 17).

Oncoplastic techniques have been demonstrated to expand the indications for breast conservation in a variety of situations (12–22). Multiple studies in recent years have shown oncoplastic breast conservation surgeries (OBS) to be as safe as BCS with superior esthetic outcomes (20, 23). Silverstein first reported on breast conservation performed in a series of cases where mastectomy was the only recommended surgical option and termed the procedure “Extreme Oncoplasty” (18). Subsequently such procedures have been shown to be a safe and viable option in selected cases with good patient reported outcomes (15, 19, 22). In a comparison of post-NACT BCS and oncoplastic volume displacement surgery, patients treated with either surgery had similar survival outcomes (24).

Here, we present an audit of our cohort of 180 breast cancer patients who were treated with post-NACT surgery, assessing breast conservation rates, oncological outcomes, and 1 year patient reported outcomes measures (PROMs). We present a series of precautions and procedures that we carried out to ensure safe breast conservation to address the lacunae/issues raised by the EBCTCG overview. At our center, we routinely perform OBS whenever needed and possible for all breast tumors, including LABC and LOBC downstaged with NACT. In recent times, the scope of patients for whom guidelines recommend neoadjuvant therapy has been broadened (25). In this scenario, evidence for the safety of post-NACT breast conservation and approaches to expand the scope of breast conservation are urgently required. Currently, although there are reports of post-NACT oncoplastic breast conservation (19, 22, 24, 26–28), most of these reports focus on a single approach for oncoplastic breast conservation. As with breast conservation, each surgical approach is appropriate only for a specific set of patients. The current study utilizes an arsenal of oncoplastic techniques and a series of precautions in a variety of scenarios to safely increase post-NACT breast conservation. In our experience, oncoplastic breast conservation has proven to be an oncologically safe procedure with good cosmetic and patient reported outcomes (19, 22). This comprehensive report on post-NACT oncoplastic breast conservation demonstrates how such techniques can be carefully leveraged to increase post-NACT breast conservation rates.

## Methods

### Patient selection

This is a single institutional study involving retrospective analysis of prospectively collected data. The following pathological criteria were used for patient selection for neoadjuvant systemic treatment: (1) LABC (not including node positive T1 and T2 LABC cases), (2) LOBC, (3) luminal HER2-positive and HER-positive (non-luminal) tumors greater than 2 cm; (4) Triple Negative Breast Cancer (TNBC) greater than 3 cm, (5) luminal HER2 negative large T2/T3 tumors, and (6) any patient with clinically or histologically proven positive axillary nodes. Patients with demonstratable metastatic disease were excluded. All patients underwent definitive breast surgery post-NACT in the period between January 2015 and December 2020. Patients were deemed qualified for upfront mastectomy based on the following criteria: (1) LABC; (2) LOBC; that is, Stage IIB and above (T3N0 but not T2N1) as described by Simos et al. (29); (3) small breast volume to tumor ratio; and (4) multicentric/multifocal tumors. Having upfront qualification for mastectomy enabled us to compute conversion rates to BCS/OBS post-NACT. The plan of management (NACT and proposed surgical plan) was discussed with the patient, and written informed consent was obtained for each step/procedure, for each patient.

### Data collection

Data included demography, medical history, clinical findings, pathological reports (diagnostic biopsy and surgical histopathology including immunohistochemistry), details of NACT, surgical intervention, pre- and post-operative images of patients, post-surgery complications, follow-up details, and PROMs.

### Clinical management

Triple assessment based on clinical examination, imaging and image-guided core needle biopsy was routinely used to establish a diagnosis. Systemic staging was assessed based on Positron Emission Tomography (PET) imaging. Patients were selected for neoadjuvant treatment based on decisions made by the multidisciplinary team (MDT). After clinical staging, NACT was administered based on NCCN guidelines.

### Tumor response

After every other cycle of chemotherapy and at the completion of NACT, the patient was monitored clinically and radiologically. Clinical response of the primary tumor to therapy was calculated according to the RECIST Ver 1.1 criteria (30). The pathological response was determined by comparing the pre-therapy clinical stage with the stage at final histopath.

### Tumor localization

**Figure 1:**
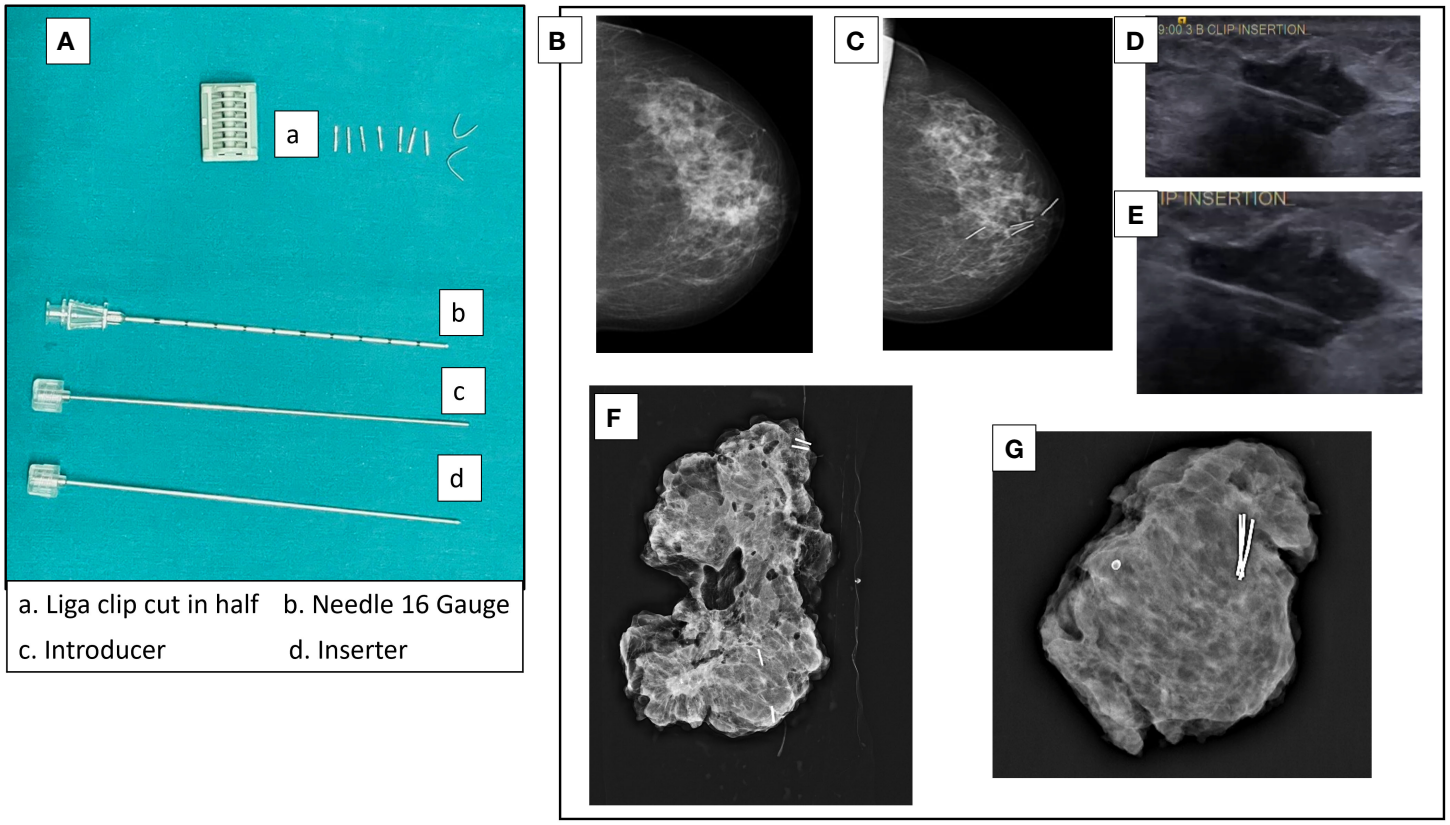
Tumor localization by liga clips mid-NACT. **(A)** Liga clips and needles used for USG-guided clip insertion. The liga clip is cut in half and deposited in the tumor mass under USG guidance with the 16-gauge needle. **(B)** Mammogram of pre-NACT lesion HER2-positive non-luminal 3 × 1.8 cm in size in upper and inner quadrant of left breast. **(C)** Mammogram of the residual lesion after two cycles of AC with clips inserted. **(D, E)** USG images of clip insertion into a tumor. **(F)** Specimen mammogram of a clipped specimen with ypT1 residual IDC + DCIS. The residual tumor was clipped at 16.5 mm on USG. The specimen is from a simple therapeutic mammoplasty procedure (volume displacement, Level 2). **(G)** Specimen mammogram of a clipped specimen with complete response (ypT0). Clipping was done when the residual tumor was 18 mm on USG. The specimen is from a complex therapeutic mammoplasty procedure (volume displacement, Level 2).

For all cases, the residual tumor was pre-operatively localized by ultrasound (USG) imaging. In cases where the tumor responded to NACT, the residual tumor was clipped mid-NACT at ~1cm size by USG guided insertion of liga clips. At least four clips were used to facilitate identification by intra-operative USG (**Figure 1**). We believe that accurate delineation of the center is difficult in a 4- to 5-cm tumor and therefore needs to be done after response to therapy and reduction in tumor volume. Mid-NACT localization after good NACT response mitigated the requirement of another localization procedure such as wire guidance before the definitive surgery. Pre-operative localization by mammography-guided wire bracketing was done only where there was extensive residual calcification post-NACT. Intra-operative USG was used to target the clipped center, and a wide excision of the clip-bearing area was carried out. Intra-operative specimen mammography and USG were used to confirm that all wire-bracketed calcifications and/or clipped specimen were excised with negative margins. **Figure 1** also shows specimen mammogram from clipped residual tumors that were T0 and T1 on final pathology.

In this report, paraffin slides of margin tissue were accessed from the pathology laboratory to verify the negative margin outcome. Slides of tissue assessed at frozen were quality checked for tissue integrity on Mantra (PerkinElmer). Frozen and pathology slides were imaged on an OS-15 (OptraSCAN, San Jose, CA, USA) bright field digital scanner. These digital images were blinded and shared from our server with the second pathologist (SB) to assess concordance in margin assessment.

### Surgical techniques

In every case, wide local excision of the residual tumor was identified by palpation as well as USG of the residual tumor and the marker clips used for localization. Post-excision margin adequacy was assessed on the table by USG or by specimen mammography. In addition, all specimens were sent for frozen section margin assessment to the pathologist. Any close margin (1–2 mm) seen on specimen mammogram or reported on frozen section (including focally positive margins) was revised in the same sitting before restoration of the breast form. Margin guidelines based on the consensus on margin safety by the American Society of Clinical Oncology, Society of Surgical Oncology, and American Society for Radiation Oncology Consensus Guideline (31) were followed.

The surgical plan was determined based on assessment at diagnosis, post-therapy clinical-radiological assessment of residual disease, residual tumor location, and extent of calcification, breast size, and ptosis. Patients were counseled for safety and esthetic outcomes. Final decisions were based on patient choice. Volume displacement and volume replacement techniques were employed in all cases where conventional breast conservation was deemed unsuitable. In cases where breast conservation was not feasible mastectomy with immediate whole breast reconstruction was performed. Very few patients did not undergo reconstruction. The surgical procedures used are described here.

### Conventional breast conservation surgery

BCS with primary breast closure was performed in patients with small residual tumors with adequately sized breasts or tumors in favorable locations.

### Oncoplastic breast conservation

Oncoplastic surgeries have been classified based on the recent recommendations of the American Society of Breast Surgeons (32) and as expanded on by Silverstein (20). As defined in these recommendations, oncoplastic surgery refers purely to oncoplastic breast conservation surgery. Oncoplastic techniques performed were simple: Level 1, complex; Level 2, volume displacement and more complex; and Level 3, volume replacement and by perforator flap in most cases. Mini-Latissimus dorsi (LD) flaps were only used in surgeries performed prior to 2019 before perforator flaps were incorporated in routine practice.

### Mastectomy with reconstruction

In certain situations, a complete mastectomy was performed. Seventy percent cases of mastectomy were followed by immediate breast reconstruction by implant or LD flaps. Dermal sling was employed for larger ptotic breasts, which has been shown to be a safe procedure (33). For cases with small breasts, an Advanced–Lower Dermal Sling (A-LDS) was used in place of an acellular dermal matrix (34).

Post-surgical oncological management decisions regarding chemoradiation protocols were undertaken by a multidisciplinary clinical team in accordance with guidelines.

### Surgical outcomes

Surgical outcome data were recorded as minor or major based on the presence or absence of surgical complications such as seroma, delayed wound healing, fat necrosis, lymphedema, and infection and the interventions required to treat them. Major complications were those that required surgical intervention, whereas minor complications could be treated by therapy in the clinic.

### Oncological outcomes

Margin status was assessed by histopathology of frozen sections intra-operatively and paraffin section post-operatively. Patients were carefully monitored for local and distant recurrences, quarterly for the first 2 years post-surgery and then every 6 months. Suspicious symptoms or signs were assessed by appropriate imaging and histological confirmation wherever feasible.

### Patient reported outcomes measures

Patients were evaluated for patient reported outcome measures (PROMs) using the BREAST-Q questionnaire (35) at 1-year post-surgery follow-up. BREAST-Q questionnaire was offered to all patients at the completion of 1-year post-surgery follow-up, and data are presented for all patients who responded.

### Statistical methods

Data were analyzed using Fishers Exact Count Statistic (when less than five cases), chi-square test, Wilcoxon Rank Test, and Student’s t-test using the stat and BSDA (36) package in R Ver 4.0 (37). Median follow-up was calculated using the reverse KM method of Schemper and Smith (38) using the Prodlim (39) and Hmisc (40) packages in R Ver 4.0. All graphs were plotted using ggpubR (41) in R Ver 4.0. Survival parameters were calculated using the survival (42) package in R. Kaplan–Meier plots were plotted using the survminer (43) package.

## Results

### Patient cohort

One hundred eighty breast cancer patients treated with neoadjuvant therapy and operated between January 2015 and December 2020 were included in the study. **Table 1** describes the demographic and clinical features of the patients included in the cohort. The median age of the patients was 50 years (range: 23–75). Most cases (41%, 74 of 180) were luminal HER2 negative, followed by TNBC (31%, 57 of 180) and HER2 + 27% (49 of 180). HER2-positive cases comprise 21 luminal HER2-positive cases and 28 HER2-positive non-luminal cases.

**Table 1:** Demographic and clinical features of cohort.

Features	Class	N(%)
*Total patients*		180
*Age*	Average ± SD years	51.5 ± 11.3
	Median (Range)	50 (23,75)
	< 40 years	27 (15%)
	40-60 years	112 (62.2%)
	> 60 years	41 (22.8%)
Clinical stage*	IIA	25 (13.9%)
	IIB	43 (23.9%)
	IIIA	71 (39.4%)
	IIIB	35 (19.4%)
	IIIC	3 (1.7%)
	Not available	3 (1.7%)
Clinical tumor size*	T1	19 (10.6%)
	T2	113 (62.8%)
	T3	20 (11.1%)
	T4	24 (13.3%)
	Not available	4 (2.2%)
Clinical node status**	Positive	154 (85.6%)
	Negative	23 (12.8%)
	Not available	3 (1.6%)
*Subtype*	Luminal HER2 negative	75 (41.7%)
	HER2-positive non-luminal	28 (15.6%)
	Luminal HER2 positive	21 (11.6%)
	TNBC	56 (31.1%)
*Tumor type*	IDC (post-excision biopsy)*†	3 (1.7%)
	IDC	156 (86.7%)
	IDC + DCIS	9 (5.0%)
	IDC + ILC	2 (1.1%)
	ILC	7 (3.9%)
	Other	3 (1.6%)
*Tumor grade*	I	3 (1.7%)
	II	111 (61.7%)
	III	53 (29.4%)
	Not available	13 (7.2%)

Demographic distribution of NACT cohort. *Clinical tumor size was assigned based on pre-NACT radiological and clinical assessment of tumor.

**Node status was assigned based on ultrasound observations and biopsy/FNAC data where available. These were used to assign a clinical stage.

*†Three cases that presented post an excision biopsy have been included. All had IDC with positive margins on biopsy histopathology, and two cases had a palpable node on presentation at our clinic. These cases were treated with neoadjuvant therapy prior to definitive surgery.

A majority of cases (86%) were diagnosed at biopsy as IDC (155 of 180), and the rest were IDC with DCIS (9 of 180, 5%) and ILC (7 of 180, 4%). Most tumors were T2 at presentation (63%, 113 of 180), and 85% of patients were node positive (153 of 180). Most of these cases were N2 on imaging (56%, 86 of 153). A significant number of cases were clinically stage IIIA at presentation (38%, 69 of 180). The three cases that were treated with neoadjuvant therapy post an excisional biopsy performed at other centers. Excision biopsy is performed on lumpectomy specimens. These are the results of surgical procedures performed for diagnosis, immediately after a suspicious imaging report or clinical examination, without any initial biopsy. This is a common practice in many centers. Such patients often come to our center for reconstructive surgery to regain breast shape, remove scars, and so forth. This is a common practice in many centers. Such patients often come to our center for reconstructive surgery to regain breast shape, remove scars, and so forth. Since margin positivity is often not assessed after such procedures, patients are always assessed for residual tumor by imaging or a histopathological review of specimen tissue when available. The three cases included here came to us post such a procedure from other centers with reported positive margins. Two of these cases also had positive nodes that had not been treated. The cases included in this cohort were young age (< 40 years) and offered NACT prior to surgery.

### Neo-adjuvant treatment

Among patients with luminal HER2-negative tumors, 49% (36 of 74) received hormonal therapy and 51% (38 of 76) were given Anthracycline- or Anthracycline- and Paclitaxel-based chemotherapy. Patients on hormone therapy were significantly older (mean age: 61 + 9.6, (range: 41–75 years) than the chemotherapy group (mean age: 48 + 10.3, (range: 29–67 years), *p =* 6.39e−07), and all except 1 were post-menopausal. Seventy-seven percent (38 of 49) of HER2-positive (luminal and non-luminal) patients received trastuzumab, a very high rate in an Indian setting for the use of HER2-directed therapy due to the prohibitive cost involved (44). Fifty-eight percent of TNBC patients (33 of 56) received AC followed by a Taxane, and the rest were treated with protocols such as AC alone, Fluorouracil, Epirubicin, Cyclophosphamide (FEC) with Taxane and Taxane with platinum drug as per guidelines. These variations in drug regimen are unavoidable confounders in our data and are a result of the socio-economic realities that patients face during treatment. In some cases where the delay in surgery and/or increased costs due to neoadjuvant therapy proved unacceptable, the preferred regiment of AC + taxane was cut down to four cycles of AC alone.

### Response to treatment and surgery

**Figure 2:**
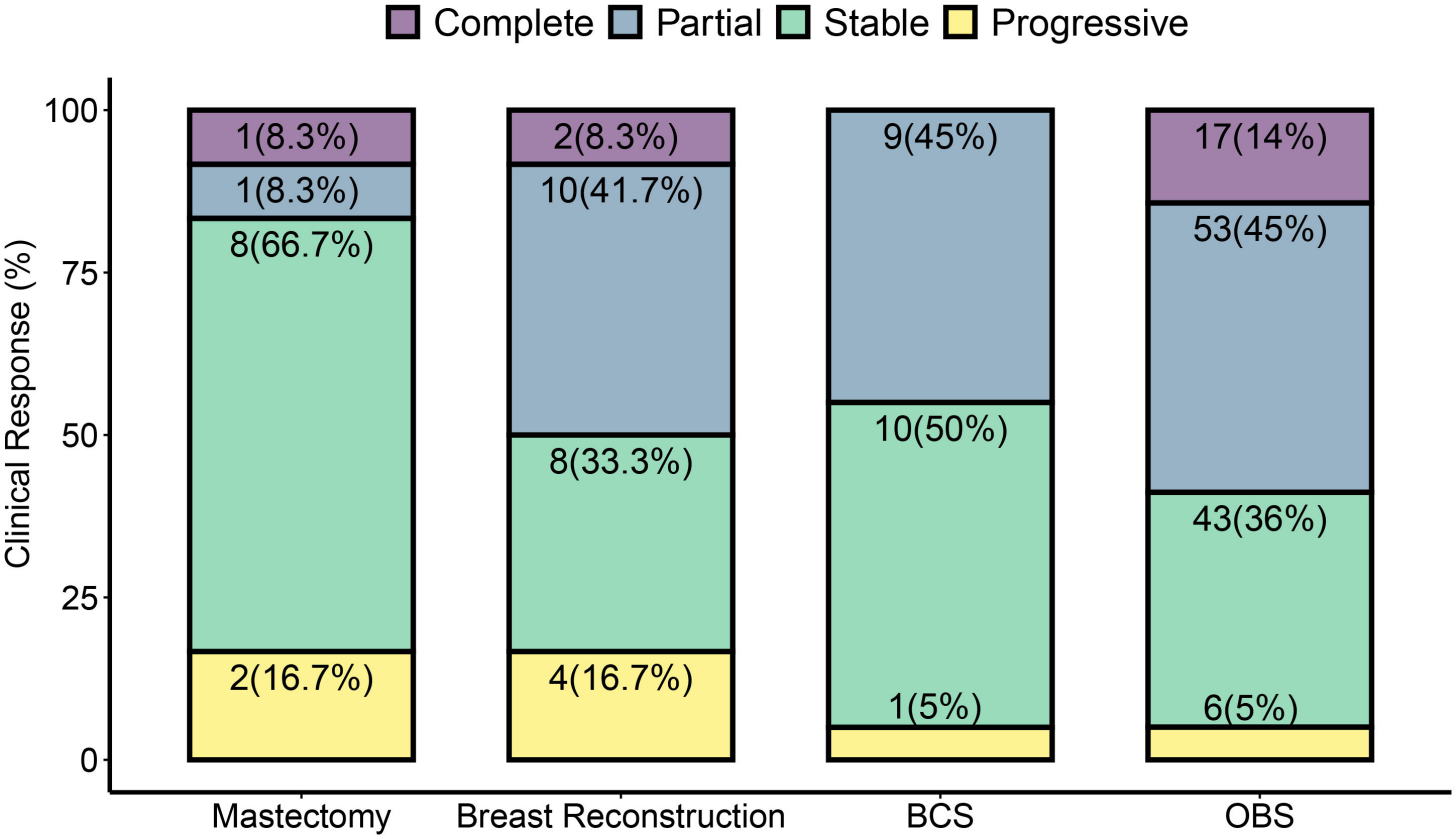
Surgery types and clinical tumor response to NACT. Post-NACT surgery type is dependent on the clinical response observed. Clinical response was determined to be complete, partial, stable, or progressive by pre-operative imaging according to RECIST criteria. A greater than 30% increase was taken as progressive disease and greater than 20% decrease as a partial response. Tumors that did not sufficiently decrease to be classified as a partial response or increase enough for progressive response were classified as stable. Cases that did not show any residual breast tumor on imaging were classified as complete response. Surgery types are classified as described in methods. Each bar represents the percent of each surgery type performed for the given clinical response type. In some cases, pre-surgical imaging was unavailable—one in mastectomy, one in breast reconstruction, two cases of OBS, and one case of BCS, and these cases are therefore omitted from this figure. Each subsection is labeled with the total number of surgeries shown in the figure. Numbers in parenthesis are % values for the response type in each surgery type.

OBS dominated as the choice of surgery in our cohort (**Figure 2**). Seventeen of 20 patients who had complete clinical response underwent OBS, while 53 of 73 patients with partial response were treated with OBS. Patients with progressive disease had the highest percent of mastectomy with immediate breast reconstruction (31%) or without reconstruction (9%).

**Figure 3:**
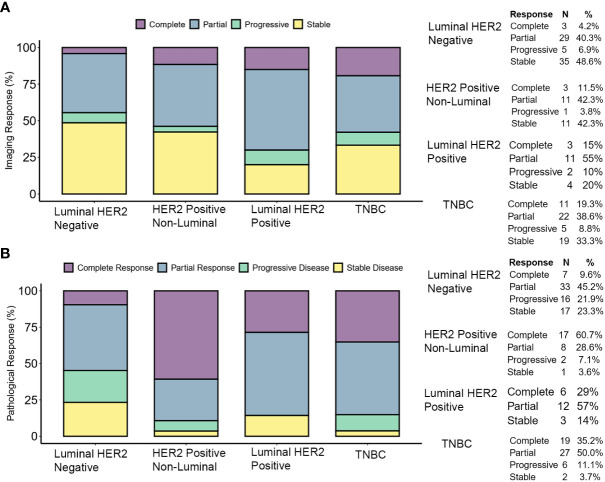
Tumor response to therapy. **(A)** Clinical response determined by pre-operative imaging. The response was determined by comparing the longest dimension of the tumor as reported by pre-operative (i.e., post-therapy) imaging versus the longest dimension reported by the same methods at the time of diagnosis. **(B)** Pathological response was determined by comparing the pre-therapy clinical stage (cTcN) to the post-therapy pathological stage (ypTypN). Complete responses shown here are ypT0ypN0 (pCR).

The clinical response to treatment was determined by RECIST criteria Ver 1.1. (30). The pattern of response based on molecular subtype is similar to the reported data (45). Complete response (pathological) was high in TNBC and HER2-positive non-luminal (pCR in 48 and 60%, respectively), and low in luminal HER2-positive and luminal HER2-negative (pCR in 29 and 10% cases, respectively). The clinical and pathological response is shown in **Figure 3**.

### Mastectomy to BCS conversion rates

Patients were classified as qualified for upfront BCS (99 of 180) or upfront mastectomy (81 of 180). The basis for qualification for mastectomy is summarized in **Table 2**. Definitive surgery was performed at the end of the prescribed NACT period, based on the clinical response of the tumor to NACT (**Figure 2**).

**Table 2:** Upfront mastectomy qualification (***n*** = 81).

Reason for qualification	N	%
**≥ 3-cm tumor in small-sized breast**	**16**	**20%**
**≥ 4-cm tumor in moderate-sized breast**	**13**	**16%**
**LABC**	**23**	**28%**
**LOBC**	**20**	**25%**
**Multicentric/multifocal**	**9**	**11%**

Basis for classification of upfront mastectomy qualification.**(A)** Cases were labeled as qualified for upfront mastectomy based on *^#^tumor size and spread (LABC/LOBC), ^†‡^Smaller tumors with high tumor to breast ratio and multicentric, multifocal tumors. One case with a small invasive tumor but extensive DCIS component has been included. LABC and LOBC were assigned as described by (27).

Out of 81 cases that initially qualified for mastectomy, 59 showed a good response to NACT and hence were converted to BCS/OBS with the conversion rate from mastectomy to BCS/OBS of 72.8%. The rest underwent mastectomy with reconstruction (15) or mastectomy alone (7) (see **Table 3** and **Supplementary Table S1**). These patients received mastectomy despite being pre-therapy candidates for breast conservation. Two of these patients had progressed on treatment, and two patients had a complete response but missed mid-NACT USG examination for clip placement and therefore had to be treated with mastectomy. For the rest of the cases, the surgery choice was based on patient preference. In some of these cases, after inadequate response to therapy and with worrisome biology, the patients were not convinced of the safety of breast conservation and were therefore treated with a mastectomy.

**Table 3:** Surgery types in the entire (***n*** = 180) and mastectomy qualified (***n*** = 81) cohort.

Surgery	Entire cohort (n = 180)		Mastectomy qualified (n = 81)	
*Mastectomy*	13	7.2%	7	8.6%
*Breast reconstruction*	25	13.9%	15	18.5%
*BCS*	21	11.7%	7	8.6%
*OBS*	121	67.2%	52	64.2%

Surgery types performed in the mastectomy qualified and entire cohorts. The mastectomy qualified cases are the cases identified in **Table 2**. Breast conservation (conventional and oncoplastic) accounts for 78.9% cases in the entire cohort and 72.8% in the mastectomy qualified subset. 72.8% is the rate of conversion from mastectomy to breast conservation.

### Post-NACT oncoplastic—breast-conserving surgery

**Figure 4:**
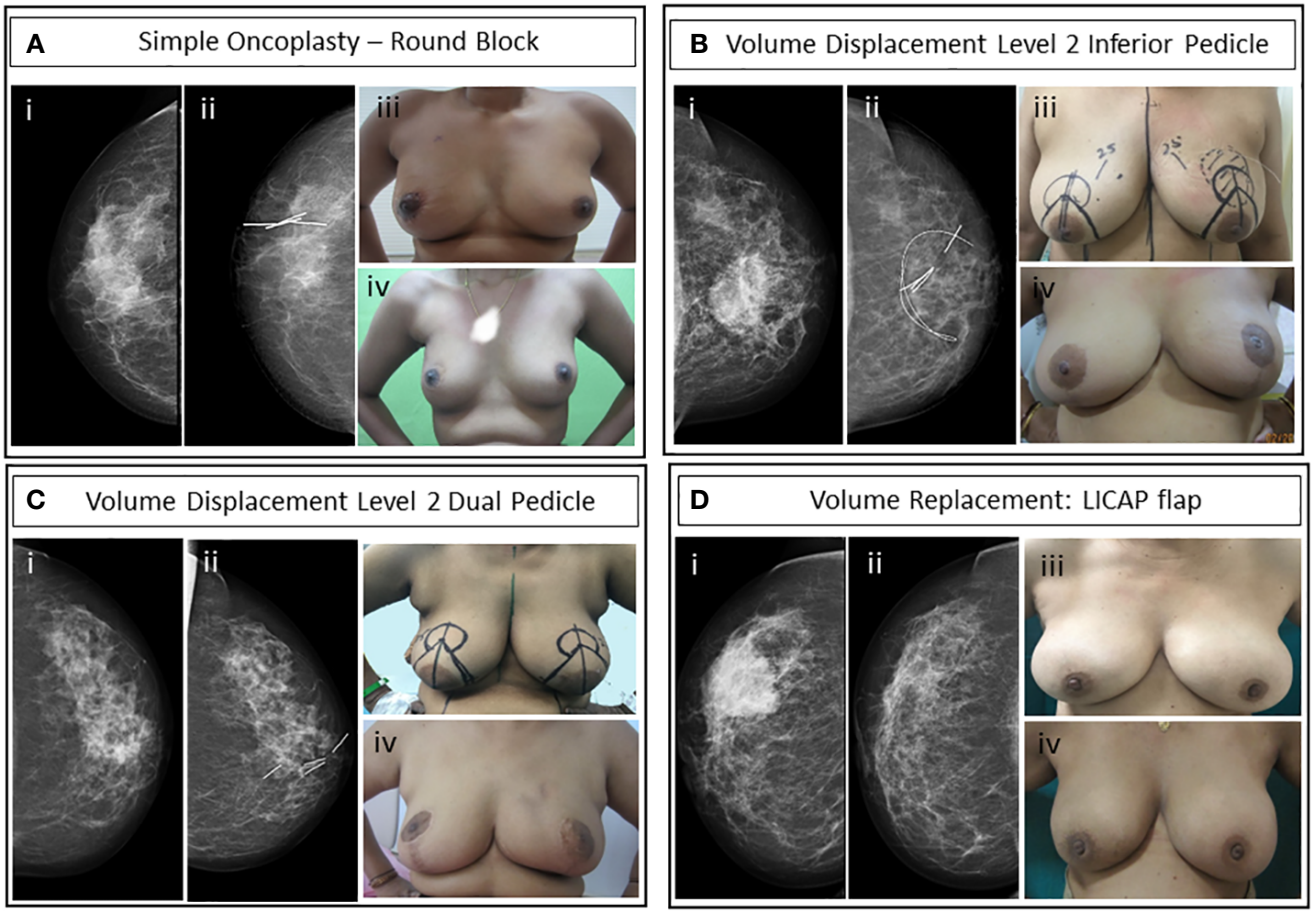
Case studies of representative OBS techniques. **(A)** Simple oncoplasty—round block incision surgery of post-NACT HER2-positive non-luminal grade II invasive breast carcinoma **(i)** pre-NACT mammogram, **(ii)** post-NACT CC view of right breast with liga-clip localization, **(iii)** immediate post-operative, and (iv) 3-year post-operative upright patient image. **(B)** Volume displacement Level 2: Inferior pedicle therapeutic mammoplasty with contralateral symmetrization reduction mammoplasty. Luminal HER2-positive grade II IDC after one cycle of NACT with paclitaxel intolerance. Bracketing wires were placed for residual microcalcifications localization. **(i)** Pre-NACT mammogram, **(ii)** post-NACT mammogram left CC view with wire localized area of residual microcalcifications and clip localized residual tumor, **(iii)** patient with pre-operative markings and wire localized tumor for left therapeutic mammoplasty and right reduction mammoplasty, and (iv) 8-month post-operative and post-RT upright patient image. **(C)** Volume displacement Level 2: Dual pedicle therapeutic mammoplasty with NAC graft and contralateral symmetrization reduction mammoplasty. Post-NACT HER2-positive non-luminal grade II IDC. **(i)** Pre-NACT mammogram CC view, **(ii)** post-NACT mammogram left CC view shows a residual lesion with marker clips, **(iii)** upright patient with pre-operative markings, and (iv) upright patient’s image 15-month post-operative. **(D)** Volume replacement: Partial breast reconstruction with LICAP flap of post-NACT grade II TNBC IDC. **(i)** Pre-NACT mammogram CC view, **(ii)** post-NACT mammogram CC view of the lesion, **(iii)** pre-operative upright patient image, and (iv) 7-month postoperative image.

Overall, 142 (80%) breast-conserving surgeries were performed in the cohort. A substantial proportion (79%) of the breast conservative operations is oncoplastic procedures (OBS) (121 of 142). The types of OBS carried out in the cohort and for the subset that qualified for upfront mastectomy are shown in **Table 4**. The frequency and type of oncoplastic surgeries in the mastectomy qualified cohort (81 of 180) and those amenable to upfront BCS (99 of 180) are not significantly different. The complexity of oncoplastic surgeries varied from volume displacement techniques (Level 1 techniques) such as rotational mammoplasty to more complex skilled procedures for extreme oncoplasty such as therapeutic mammoplasty and perforator flaps. Volume replacement procedures (46 of 142) following partial mastectomy utilized perforator flaps in most cases (83%, 38 of 46) and for the rest mini-LD (17%, 7 of 46) was performed, if such procedures could achieve superior outcomes. Mini-LD partial reconstructions were mostly done before 2019 when we had yet to adopt perforator flap as a routine technique in our practice. Examples of imaging findings and oncoplastic procedures are illustrated in **Figure 4**.

**Table 4:** Oncoplastic breast conservation techniques used.

Oncoplastic surgery type and subtype	NAT cohort	Upfront mastectomy
	N (%)	N (%)
*Volume displacement: Level 1 - Round block technique*	14 (11.6%)	4 (7.7%)
*Volume displacement: Level 1 - Lateral mammoplasty*	10 (8.3%)	2 (3.8%)
*Volume displacement: Level 1 - Simple oncoplastic closure*	6 (5.0%)	1 (1.9%)
*Volume displacement: Level 1 - Rotational mammoplasty*	2 (1.7%)	2 (3.8%)
*Volume displacement: Level 1 - Wise pattern incision*	2 (1.7%)	2 (3.8%)
*Volume displacement: Level 1 - Grisotti flap*	1 (0.8%)	-
*Volume displacement: Level 1 - Medial mammoplasty*	1 (0.8%)	1 (1.9%)
***Volume displacement Level 1***	**36 (29.8%)**	**12 (23.1%)**
*Volume displacement: Level 2 - Therapeutic mammoplasty: Simple*	17 (14%)	7 (13.5%)
*Volume displacement: Level 2 - Therapeutic mammoplasty: Extreme*	8 (6.6%)	6 (11.5%)
*Volume displacement: Level 2 - Therapeutic mammoplasty: Complex*	13 (9.1%)	2 (3.8%)
*Volume displacement: Level 2 - Therapeutic mammoplasty: Type unavailable*	2(1.7%)	2 (3.8%)
***Volume displacement: Level 2***	**40 (33.1%)**	**17 (32.7%)**
***Volume replacement: mini-LD flap***	**7 (5.8%)**	**4 (7.7%)**
***Volume replacement: Perforator flap***	**38 (31.4%)**	**19 (36.5%)**
***Total cases***	**121 (100%)**	**52 (100%)**

Distribution of oncoplastic breast conservation techniques used in post-NAST surgery. Level 1 surgeries were of a wide variety with reduced excision volumes (**Figure 4**). Volume replacement surgeries by perforator flaps were mostly LICAP (n = 26/40) and included TDAP, LTAP, MICAP, Epigastric thoracic flap, and two cases of LICAP with an LTAP flap. Text in bold is a summation of the previous data and scores corresponds to how the levels of oncoplastic surgery are related to surgery.

*Two cases of therapeutic mammoplasty have not been classified as simple, complex or extreme, and have therefore not been included in the details.

Patients who were selected for volume displacement Level 2 surgeries were also counseled for contralateral symmetrization. Only patients who consented for symmetrization were treated with this oncoplastic technique (40 cases). In addition, three cases of perforator flap volume replacement and 11 cases of immediate breast reconstruction received simultaneous symmetrization.

**Figure 5:**
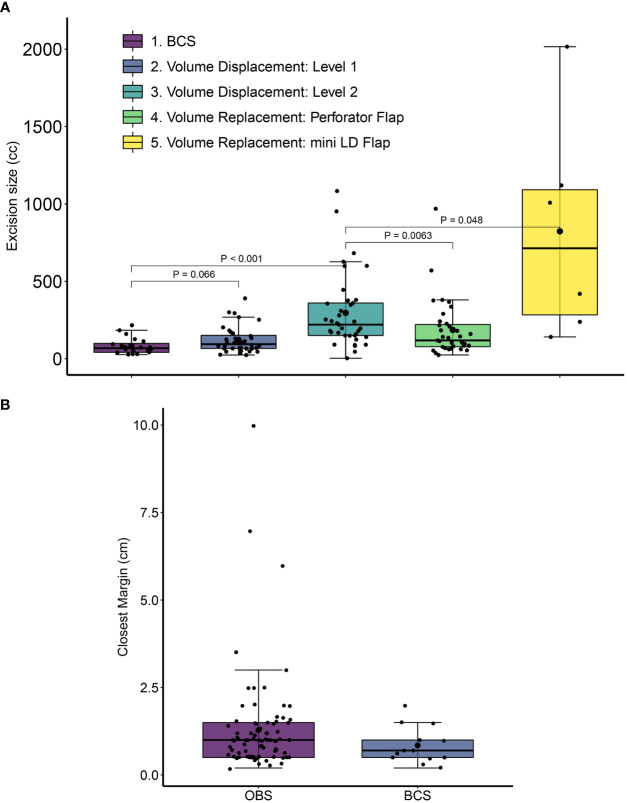
Excision volumes and closest margin distance for post-NACT breast conservative surgery. **(A)** Excision volumes were calculated from the sum of the specimen volume and the volume of the cut margins. Error bars represent standard deviation values. *P*-values are from Wilcoxon Rank Sum tests with 95% confidence intervals. Error bars represent standard deviation values. **(B)** The distance of the closest margin from residual tumor at final pathology is shown for conventional breast conservative (BCS) and oncoplastic breast conservative surgeries (OBS) in the cohort. All distances are in cm. For OBS margins the closest margin is calculated after considering revised margin dimensions. Margin data were available for 13 BCS and 83 OBS cases.

The tissue volume after resection was significantly smaller in BCS (82 ± 53 cc) as compared with average resection volume in OBS procedures (235 ± 280 cc, *p* = 0.0012) (**Figure 5A**). To ensure the oncological safety of breast conservation, larger volumes of excisions were required in some cases; hence, we had to adopt OBS techniques. The volume and the location of the excision required dictated the complexity of the OBS technique required (**Figure 5A**).

### Adjuvant treatment

A total of 40% patients (72 of 180) received adjuvant chemotherapy. A majority of HER2-positive (luminal and non-luminal) patients received adjuvant trastuzumab (57%), while only 45% (25 of 56) TNBC patients and 49% (36 of 74) luminal HER2-negative patients received adjuvant chemo- or hormonal therapy. Most patients received adjuvant radiotherapy (RT) (92%—167 of 180). Of the patients who did not receive RT, seven were lost to follow up, and six patients refused RT. Of these six patients, none had recurred at the time of last follow-up (average follow-up for these patients = 39 months).

### Post-surgical complications

The frequency and type of post-surgical complications observed in the NACT cohort in the first year post-surgery are shown in **Table 5**. Minor complications including seroma, fat necrosis, and insignificant delayed wound healing in OBS surgeries were observed in 19% of cases (23 of 121) and in 16% of immediate breast reconstruction surgeries (4 of 25). Two patients had major complications due to delayed wound healing post and OBS surgery, leading to flap necrosis and completion mastectomy in one case and abdominal flap graft in the other. Delayed wound healing in one case of breast reconstruction led to loss of implant (more than 1 year post-surgery). All other complications were minor and were treated with either no intervention or conservatively without needing to go back to the theater.

**Table 5:** Early surgical complications observed in NACT cohort (at 1-year post-surgery).

Complication type	Surgery type			
	Mastectomy	Breast reconstruction	BCS	OBS
*Delayed wound healing*	–	1	–	7 + 2*
*Haematoma*	-	-	-	1
*Seroma*	–	2	1	6
*Lymphedema*	1	-	-	-
*Fat necrosis*	–	1	–	6
*Hematoma*	-	-	-	1
*Capsular contracture*	–	–	–	–
*Complication rate %(n)*	7.7% (1/13)	16% (4/25)	4.8% (1/21)	19% (23/121)

Surgical complications: Post-operative surgical complications in the NACT cohort at 1-year post-surgery. Complications are described as major or minor depending on the intervention required. The rate of complications in oncoplastic breast conservation versus breast reconstruction was not significantly different. *Major complications.

### Oncological outcomes

#### Surgical margins

We assessed margin positivity rates and the minimum tumor margin size in BCS and OBS surgeries. Due to the extreme care during the surgical procedure with stringent radiological and pathological analysis and revision of margins in the same setting (see Methods), none of our patients needed a second surgery. To independently verify intra-operative frozen assessment of negative margins, pathology margin slides were imaged, blinded, and shared with an independent US-board certified pathologist (SB) for assessment of margin involvement. One hundred sixty-two slides (68 specimen margins, 46 revised margins, 43 frozen specimen margins, and five frozen-revised margins) from 23 patients were re-examined. All the examined margins were found to be negative. In patients with scattered tumor in excised tissue, much wider than 2-mm margins were taken to ensure not to miss any scattered residual disease. The average margin in breast conservation surgeries was 12.2 ± 13.4 mm, with larger average margins for OBS compared with BCS (12.8 ± 14.3 mm compared with 8.5 ± 5.3 mm). **Figure 5B** shows the spread in tumor margins for breast conservative surgeries (OBS and BCS) in the cohort. There were no cases of a positive margin on final pathology. In 15 cases (10.5%), margins were found to be involved or close intra-operatively (≤ 2 mm) on frozen and were revised in the same surgery. The revised re-excision margins were free of tumor on frozen and final pathology.

### Survival outcomes

**Figure 6:**
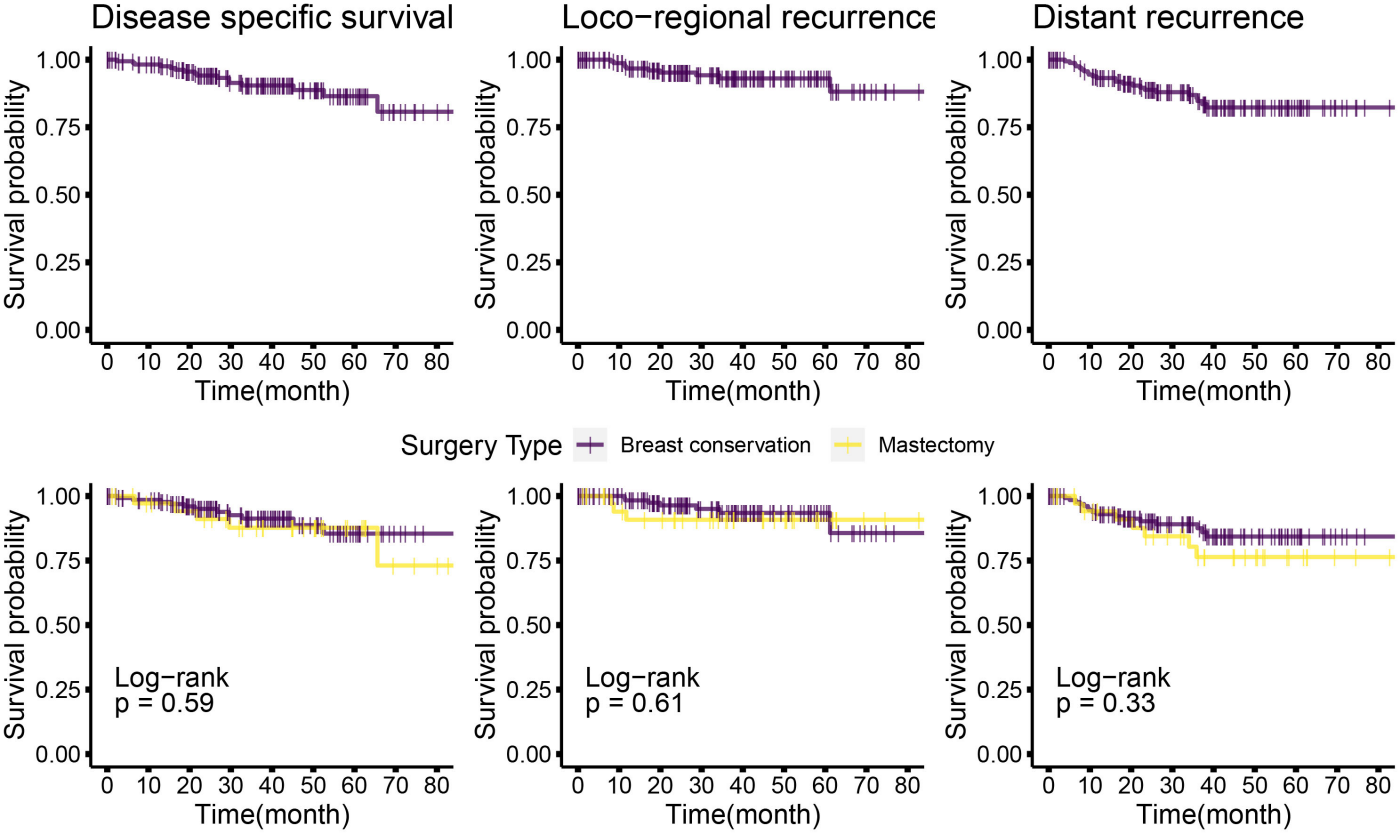
Kaplan–Meier plots of disease-specific, distant disease–free, and locoregional disease–free survival for the entire cohort, and mastectomy or breast conservation treated subsets. Top panel: Kaplan–Meier plots for the entire cohort for different survival types; bottom panel: Kaplan–Meier plots for the mastectomy and BCS-treated subsets. Log rank *P*-values are shown; survival types: The left most plots show the disease specific survival; central panel shows the plots for locoregional disease–free survival and the right most are for distant disease–free survival. Disease-free interval is calculated from the time of surgery to the first recurrence (local or distant) observed. OS time is calculated from the time of surgery to the most accurately available date of death where the death is known to relate to cancer. In cases where date of death was not available, the date of last follow-up has been used. Distant disease–free survival is plotted for the time from surgery to a distant recurrence or detection of systemic metastasis. Distant recurrence may be the first recorded recurrence or subsequent or concomitant to a local recurrence. Similarly, loco-regional recurrences shows data for the first recorded recurrence or concomitant with a distant recurrence.

**Figure 7:**
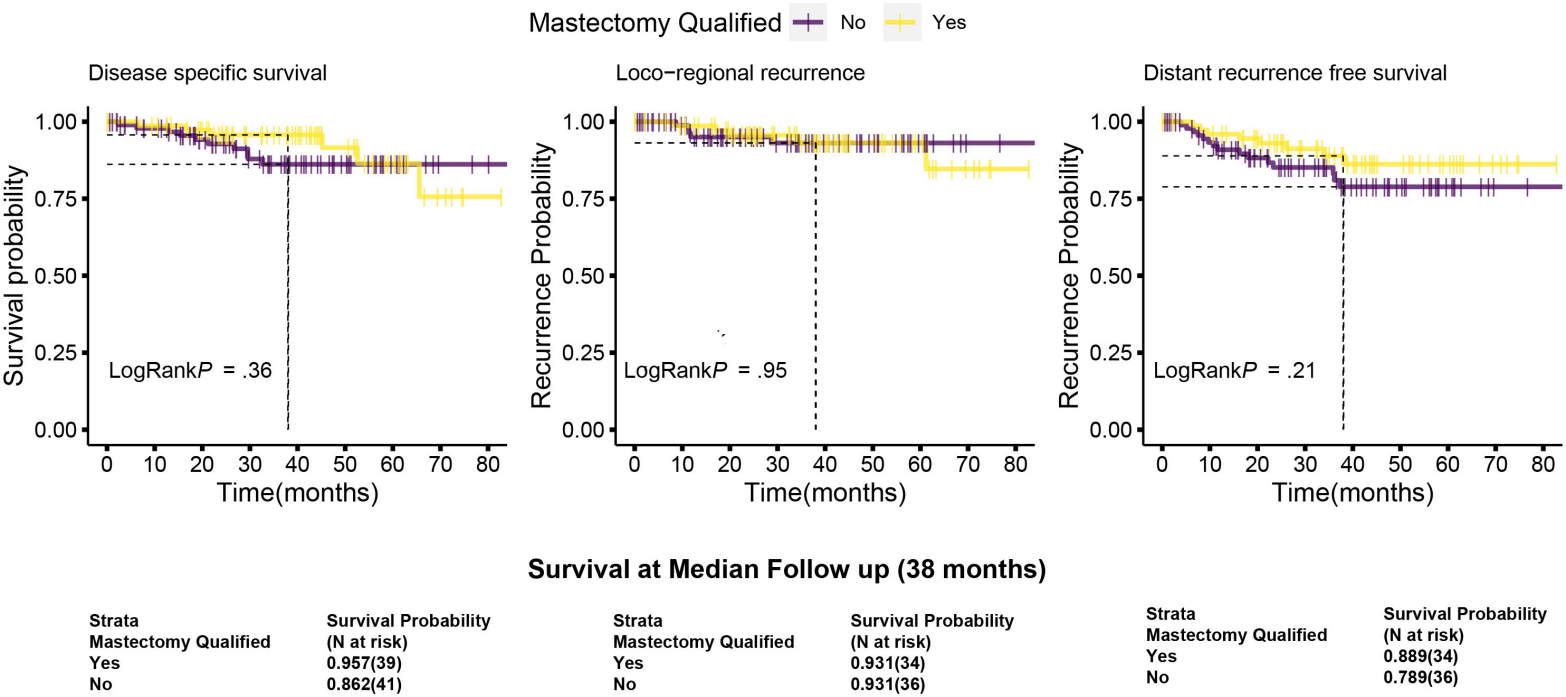
Kaplan–Meier plots of disease-specific, distant disease–free and locoregional disease–free survival for the mastectomy qualified and unqualified subsets. Survival types: The left most plots show the disease-specific survival; central panel shows the locoregional disease free survival and the right most are for distant disease free survival. Dotted lines indicate the survival probabilities at median follow-up stratified by mastectomy qualification and log-rank *p*-values (at 95% confidence interval) for each survival type. Survival is calculated as described in **Figure 6**. Survival probabilities at median follow-up are given in the table below with number at risk. Data are taken from the actuarial tables used to plot the survival curve.

The median follow-up for this cohort is 38 months (minimum 6.1 months and maximum 86 months, i.e., 7.2 years). Sixteen cases (9%) have less than 1-year follow-up. At median follow-up (38 months), the entire cohort had an overall survival (OS) rate of 90.5%, distant disease–free survival of 84%, and loco-regional recurrence free survival at 93% (**Figure 6** upper panel and **Table 6**). The 142 breast conservation–treated cases had an OS rate of 91.3%, distant disease free at 86.0%, and LRR-free survival of 93.5%. (**Figure 6** lower panel). These are acceptable rates for post-NACT–treated cohorts. The Kaplan–Meier plot for surgery stratified disease free and OS is shown in **Figure 6**. Surgery-type specific local and distant recurrences that occurred during median follow-up time are shown in **Table 7**. Further details for each breast conservation type are shown in **Supplementary Table S2**. Cox proportional hazards showed no significant difference at median follow-up (38 months) between breast conservation (conventional BCS and OBS) and mastectomy (mastectomy with or without immediate breast reconstruction) for both distant and local disease free and OS (data not shown). This pattern was seen even in the comparison of the outcomes for the mastectomy qualified versus unqualified cohort (**Figure 7**). Subtype-stratified distant and local disease free and OS was significantly worse for TNBC subtype. Multivariate cox proportional hazards showed that only TNBC subtype and pathological node status are significantly associated with disease free outcomes in this dataset (data not shown).

**Table 6:** Survival outcomes at 3 years (median follow-up) post-surgery local, distant recurrence, and survival percentages.

Follow up status	Mastectomy N = 13	Breast reconstruction N = 25	BCS N = 21	OBS N = 121
At median follow-up (3-year post-surgery)				
*Local recurrence*	3 (23%)	0	0	7 (6%)
*Distant recurrence*	1 (8%)	6 (24.0%)	5 (24%)	10 (8%)
*Death due to Cancer*	2 (15%)	2 (8.0%)	1 (5%)	8 (7%)
*Death due to unrelated cause*	-	-	1 (5%)	-

Survival outcomes: Crude survival and recurrence rates at median follow-up (3 years) post-surgery. Outcomes are shown according to surgical treatment received. Numbers represent the number of events for each survival type that occurred before median time point. Percentages are calculated from the total number of surgeries of the given type performed.

**Table 7:** Kaplan–Meier survival estimates*.

Time (months)	NACT cohort	Mastectomy	Breast conservation	Log rank p
Disease-specific survival				
*38*	0.904(80)	0.877 (22)	0.913 (59)	0.59
Distant disease–free survival				
*38*	0.835(70)	0.763 (17)	0.860 (54)	0.33
Locoregional recurrence–free survival				
*38*	0.931(70)	0.908(17)	0.935(54)	0.61

Kaplan–Meier estimate of disease (local and distant)-free and overall survival. Survival probabilities for the entire cohort and stratified by surgery type (mastectomy and breast conservation) are shown with log-rank p-values (at 95% confidence interval) for the comparison between mastectomy and breast conservation outcomes. Survival is analyzed for overall survival, distant disease–free survival, and locoregional recurrence–free survival. Data are taken from the actuarial tables used to plot **Figure 6**.

### PROMs

The PROM scores from the BREAST-Q questionnaire (35) (Questions 1–5) are shown in **Table 8**. The data represent 1-year PROM scores collected for 18 of 25 patients who had a mastectomy with immediate breast reconstruction, and 72 of 121 patients who underwent OBS (22 volume displacement Level 1, 25 volume displacement Level 2, and 25 volume replacement—perforator flaps). All parameters are satisfactory in all three surgery types, that is, breast reconstruction, BCS, and OBS (values range from 67% to 88%). The variation among surgery types for scores of Question 1 (satisfaction with breasts) was significantly different in ANOVA analysis. OBS showed significantly better satisfaction scores compared with reconstruction, for Question 1 (**Table 8**) (*P* < 0.001) by Mann–Whitney Test.

**Table 8:** Patient-reported outcome measures at 1-year follow-up.

Breast-Q question	Breast^†^ reconstruction	BCS^†^	OBS^†^	**p
1.Satisfaction with breasts	68 ± 16 (16)	80 ± 19 (7)	81 ± 14 (72)	0.0046
2.Satisfaction with outcome	88 ± 16.6 (16)	73 ± 14.7 (7)	84 ± 9.5 (72)	0.0188
3.PsychoSocial well-being	83 ± 20 (16)	86 ± 20 (7)	87 ± 17 (72)	0.66 (N.S)
4.Sexual well-being	52 ± 37 (5)	76 ± 22 (5)	80 ± 25 (34)	0.11(N.S)
5.Physical well-being	72 ± 17 (16)	68 ± 6 (7)	73 ± 13 (72)	0.63 (N.S)
**Response rate**	**16/25 (67%)**	**7/21 (64%)**	**72/121 (80%)**	

Patient-reported outcomes measure. Mean scores for patient response to selected Breast-Q questions. The number of patients who responded varied by question and is given in parenthesis.The total rate of response for each surgery type is given as response rate (bold) at the bottom of the table.

## Discussion

Breast conservation gives the best surgical outcome for breast cancer in appropriately selected patients (15). Recently, BCS has been shown to be associated with better survival outcomes in early breast cancer compared with mastectomy (46, 47) in data from the Dutch national registry (T1-T2, N0-N2), and the Danish National Registry cohort (pT1-3/pN1-3), (47) even after adjusting for confounding factors such as age, tumor size, and treatment. Conventionally, mastectomy is considered the safe surgical option for LABC/LOBC disease regardless of the response to NACT (15). Encouraged by the establishment of BCT as a safe treatment, breast conservation in LABC patients with good response to NACT has been shown to be as safe as mastectomy, with comparable LRR, disease-free survival (DFS), and OS rates (23, 48). Meta-analyses show that the rate of conversion from mastectomy to BCT post-NACT using conventional BCT techniques is around 40% globally (14, 16, 17). In the EBCTCG overview of 2018, the average conversion rate of planned mastectomy to breast conservation was 33% in the post-NACT setting. In India, this rate is still lower at 11–23% (49), with few isolated cases of higher conversion rates of up to 46.5% (50). Breast conservation gives the best surgical outcome for breast cancer in appropriately selected patients (15). Given this fact, there is a need for better utilization of this technique in the post-NACT setting.

The long-term EBCTCG overview of neoadjuvant compared with adjuvant therapy (14) did not show any specific survival benefit for NACT. Instead, NACT-treated cases had a higher rate of local recurrence. This difference was substantially reduced when cases not treated with surgery were removed from the dataset. The remaining increase in local recurrence was attributed to the increase in post-NACT BCS. The authors discuss that this increase could be a result of flaws in the included studies such as the inconsistency of imaging protocols, tumor localization, and rad-path analysis. In addition to these flaws, the assumption that post-NACT excision volumes decrease as residual tumor decrease in size (13) has led to high-positive margin and re-excision rates for post-NACT BCS (51). Conventional BCS requires small excision volumes and favors specific breast quadrants for acceptable cosmetic outcomes (12). Even post-NACT, conventional BCS, is appropriate and safe only in selected patients with a large breast to tumor volume ratio for tumors in the appropriate quadrants (14, 52, 53). In addition, even after good NACT response, large tumors may respond with a honeycomb-like residual tumor (13, 54, 55) and with residual calcifications or fibrosis, which mimic residual tumor on visual examination. Such cases would require increased excision volumes, making conventional BCS an inappropriate surgical choice.

Oncoplastic techniques have previously been shown to drastically increase the scope of breast conservation and reduce the rate of mastectomy with better margin positivity and re-excision rates (28, 56–58). Silverstein et al.(18, 59) first reported on extreme oncoplasty where patients who would be advised mastectomy in the conventional and contemporary practice were offered breast conservation with the use of oncoplastic techniques. We, in 2019, reported a series of patients with large tumors, multifocal and limited multicentric disease, post-NACT large residual tumor/calcifications, and certain extreme conditions who underwent extreme oncoplastic breast surgery (OBS) with excellent cosmesis and oncological outcomes. There are a few reports of the effective use of oncoplastic surgical techniques in post-NACT cohorts (24, 26, 28) with better outcomes with the technique. Losken and group compared simple BCS with volume displacement oncoplastic surgery in a series of late-stage (> T2 or N1) cases. Even though the group treated with oncoplastic techniques had significantly larger tumors and higher T stage, recurrence, metastasis, and survival rates were not significantly different between BCS and OBS (24).

Here, we report post-NACT outcomes for a series of 180 patients. All cases were operated by a single surgical oncologist after extensive patient counseling and with the aim of providing the best result to the patient in terms of oncological safety, long-term outcomes, cosmetic outcome, and patient satisfaction. Mastectomy has been shown to have an adverse effect on quality of life end points for most patients (5). In our practice, we aim to offer the advantage of breast conservation where oncologically safe to every patient. In India, due to a complex interplay of social norms and economic status of women, a second surgery is not an option for most patients. Hence, negative margins on the first surgery are essential. To achieve this, every tumor is widely excised and margins < 2 mm are revised. In certain situations where there is a large residual scar or lesion or scattered tumor in the excised specimen on frozen, much larger margins were taken (average margin 1.2 cm, **Figure 5B**). This may come at the expense of surgical simplicity, thus requiring complex oncoplastic techniques to achieve acceptable esthetic outcomes.

The flaws brought out by the EBCTCG overview about the inconsistency of the protocol of imaging, tumor localization, and rad-path analysis were mitigated by adherence to a strict protocol as discussed. In case of tumors that responded well to NACT, the tumor center was localized mid-NACT at ~1–2 cm by USG-guided insertion of at least four liga clips (see **Figure 1**) as discussed in methodology. The four clips used here were used to aid the identification of the residual tumor by intra-operative USG. The clips are inserted under USG guidance in the center of the residual tumor and not at the edges of the residual tumor. Accurate location of the center of the residual tumor by intra-operative USG also allows us to avoid additional procedures for wire localization. By placing the radio-opaque clips mid-NACT after a good response, as opposed to pre-NACT, we ensure that procedures such as wire localization of the clips are not routinely required (60). Only in cases where there is extensive calcification post-NACT, wire localization was done pre-operatively to identify the area of calcifications (**Figure 4B**). At times, this was in addition to prior mid-NACT clip localization of residual tumor. Other authors have used clip insertion before us; however, the purpose was to identify the tumor footprint (61). We follow the current recommendations of excision of post-NACT residual tumor rather than pre-NACT tumor footprint. This addressed the problem reported by the EBCTCG overview concerning improper localization. Two cases with a complete response missed mid-NACT follow-up and had to be treated with mastectomy since the tumor bed could not be accurately identified. Residual calcifications were targeted with bracketing and excised completely with immediate radiological confirmation. We employed intra-operative assessment of margins on frozen sections to ensure clear margins. The use of frozen sections to ensure clear margins has been reported and encouraged earlier by an Italian group of oncologists (62). This helped in ensuring zero margin positivity rates and reduced the repeat surgery rates to zero. Margin negativity was confirmed by blinded assessment by a second independent US-board certified pathologist of 100 randomly selected margin slides.

A recent meta-analysis on outcomes of post-NACT BCS (51), reported 2–33.6% positive margins with 0–12.4% re-excision rates. In contrast, in our cohort, even though 80% (142 of 180) surgeries are breast conserving, we report 0 positive margins with 0 re-excisions. In the subset that qualified for mastectomy at presentation, 73% cases (59/81) could be converted to breast conservation post-NACT. This conversion rate is mostly achieved by OBS, since 88% of breast conservation surgeries are oncoplastic surgeries (52/59). Previously, 6% positive margins were reported in a series of 47 post-NACT oncoplastic surgeries (24). Similar rates in our setting would put a strain on our system where socio-economic issues make second surgeries extremely difficult to perform. The use of frozen section removed this concern in our dataset, since we report 0 positive margins on final pathology. Stringent and extensive imaging and rad-path analysis with accurate delineation of the tumor bed were followed by well-planned radiation techniques. All these measures have resulted in excellent tumor control in the median follow-up of three years with an overall survival rate of 91.3%, distant disease free at 86% and LRR free survival 93.5% for breast conservation treated cases. These compare very well with 5 year rates reported by Chen et al. for 401 post-NACT BCT-treated patients with 63-month median follow-up (87, 89, and 91%, respectively) (48) despite the fact that conventional BCT makes up only 15% of the breast conservation surgeries (21 of 142) in our dataset. Importantly, disease-free interval is dependent on only disease characteristics such as TNBC subtype and pathological node positivity (data not shown). The type of surgical procedure used (breast conservation or mastectomy) does not affect the disease-free or survival outcomes. These results also hold true for the mastectomy qualified cohort.

The relatively short median follow-up of 38 months (~3 years) may have biased some of the oncological outcome presented here. This cohort covers 6 years of retrospective data from 2015 to 2020. The first 3 years (2015–2018) have a median follow-up of 50 months and account for 60% of the cases. Cases from 2019/2020 have shorter follow-up of 29–20 months. However, many of the patients in this cohort are in active follow-up and the outcomes of this cohort will be subsequently updated. In addition, in the time covered by this retrospective cohort, the practice was to offer the PROM questionnaire only once at the time of the 1 year follow-up. We are therefore unable to present long-term patient outcomes.

With a range of oncoplastic techniques, we were able to achieve a rate of 80% breast conservation in our cohort despite having 61% (110/180) LABC cases. Oncoplasty conferred the ability to excise a variety of volumes as was required in each case with adequate margins to make the surgery oncologically safe. These included cases where the residual tumor was large or the excised specimen sent for the frozen section showed scattered tumor foci. The esthetic outcomes were deemed acceptable based on the satisfactory PROM scores. Carefully carried out breast oncoplasty has the potential to increase breast conservation rates and patient satisfaction in the post-NACT setting. The incorporation of oncoplastic techniques helps treat larger tumors, achieves better cosmetic outcomes, and maintains comparable survival rates as that of mastectomy.

Our experience shows that meticulous protocol for imaging and targeting the tumor with mid-cycle clipping, intra-operative evaluation of margins, stringent RAD-PATH analysis, and application of appropriate oncoplastic and RT techniques confers a major benefit in terms of surgical, oncological, and patient-reported outcomes. Using these protocols, we were successful in avoiding re-excisions by a second surgery and providing breast conservation in our socio-economic conditions. In fact, avoiding second surgeries should be an aim even in the developed world as it would help save on resources. The inclusion of oncoplastic surgery in the armamentarium of surgical techniques will improve breast care for patients presenting with larger tumors and more advanced disease, which is found to a larger extent in developing countries.

## Data availability statement

The datasets presented in this article are not readily available because The dataset is composed of clinical data with follow up of individual patients treated by us and is therefore not shareable with others outside our institutions. Requests to access the datasets should be directed to dr.koppiker@prashanticancercare.org.

## Ethics statement

The studies involving human participants were reviewed and approved by PCCM-CTCR Independent Ethics Committee. The patients/participants provided their written informed consent to participate in this study. Written informed consent was obtained from the individual(s) for the publication of any potentially identifiable images or data included in this article.

## Author contributions

CK was involved in the conception and design, financial support, administrative support, manuscript writing, and final approval of the manuscript and was generally accountable for all aspects of the work; DK was involved in the collection, analysis, visualization and interpretation of data, manuscript writing, final approval of the manuscript; MK was involved in the collection, analysis and visualization and interpretation of data, manuscript writing; SK was involved in the collection, analysis and visualization of data; MP was involved in the collection and analysis of data; UD, CD, BV, NJ: Assembly of data; VZ in data interpretation and manuscript writing; NG, AJ in data collection and analysis; RU, RB, NN in data collection; PV in data analysis; LB Administrative support; GT and SN data assembly and collection; JP in data interpretation; SB in data analysis and interpretation. All authors contributed to the article and approved the submitted version.
