# Supplementary material for: Impact of oncoplasty in increasing breast conservation rates Post neo-adjuvant chemotherapy
Source: Front Oncol. 2023 Sep 7;13:1176609. doi: 10.3389/fonc.2023.1176609 (PMC10514208; doi:10.3389/fonc.2023.1176609)
Supplement: Supplementary file 1 [file DataSheet_1.pdf]

Supplementary Table 1
Mastectomy to Breast Conservation Conversion

Breast Conversion Decision	**BCS**	**OBS**	**Total Cases**	**%**
Pre-NACT	18	69	99	0.88
Post-NACT	7	52	81	0.73

Distribution of surgery types used for treatment in upfront mastectomy qualified cohort (n=81) and in the rest of the NACT cohort (n=99).

Supplementary Table 2
Local and Distant Recurrences in Breast Conservative Surgery

Breast Conservative Surgery Type	**Loco-Regional**		**Distant**	
	N (%)	Time (months)	N (%)	Time (months)
Conventional BCS (N = 21)	-	-	5(24%)	4-26
Volume Displacement: Level 1 (N = 36)	3(8.3%)	11-61	3(8.3%)	5-38
Volume Displacement: Level 2 (N = 40)	1(2.5%)	3	5(12.5%)	8-22
Volume Replacement: Perforator Flap (N =38)	4(10.5%)	11-34	3(7.9%)	10-36
Volume Replacement: mini-LD (N =7)	-	-	1	37
